# Effects of the Histone Deacetylase Inhibitor Valproic Acid on Human Pericytes *In Vitro*

**DOI:** 10.1371/journal.pone.0024954

**Published:** 2011-09-22

**Authors:** Jakob Karén, Alejandro Rodriguez, Tomas Friman, Lennart Dencker, Christian Sundberg, Birger Scholz

**Affiliations:** 1 Department of Medical Biochemistry and Microbiology, Uppsala University, Uppsala, Sweden; 2 Department of Pharmaceutical Bioscience, Uppsala University, Uppsala, Sweden; 3 Department of Women and Children's Health, Uppsala University Hospital, Uppsala, Sweden; Pennington Biomedical Research Center, United States of America

## Abstract

Microvascular pericytes are of key importance in neoformation of blood vessels, in stabilization of newly formed vessels as well as maintenance of angiostasis in resting tissues. Furthermore, pericytes are capable of differentiating into pro-fibrotic collagen type I producing fibroblasts. The present study investigates the effects of the histone deacetylase (HDAC) inhibitor valproic acid (VPA) on pericyte proliferation, cell viability, migration and differentiation. The results show that HDAC inhibition through exposure of pericytes to VPA in vitro causes the inhibition of pericyte proliferation and migration with no effect on cell viability. Pericyte exposure to the potent HDAC inhibitor Trichostatin A caused similar effects on pericyte proliferation, migration and cell viability. HDAC inhibition also inhibited pericyte differentiation into collagen type I producing fibroblasts. Given the importance of pericytes in blood vessel biology a qPCR array focusing on the expression of mRNAs coding for proteins that regulate angiogenesis was performed. The results showed that HDAC inhibition promoted transcription of genes involved in vessel stabilization/maturation in human microvascular pericytes. The present *in vitro* study demonstrates that VPA influences several aspects of microvascular pericyte biology and suggests an alternative mechanism by which HDAC inhibition affects blood vessels. The results raise the possibility that HDAC inhibition inhibits angiogenesis partly through promoting a pericyte phenotype associated with stabilization/maturation of blood vessels.

## Introduction

Microvascular pericytes are cells of mesenchymal origin situated juxtaposition to the endothelial layer in the microvasculature *i.e.* capillaries, venules and small arterioles. They are continuous with the vascular basement membrane. Pericytes have a central role in the structural and functional integrity of the microvascular bed in resting tissues. Their equivalents in larger vessels are smooth muscle cells [1]. During development and in adult activated tissues they are important modulators of the angiogenic process where they regulate vascular regression, pruning and vessel maturation during tissue remodeling [2]. Pericytes also play a role in promoting platelet aggregation [3]. Thus pericytes, in addition to endothelial cells, must also be tightly controlled in order to maintain tissue homeostasis, optimize tissue repair and regeneration. Their role in tissue repair is further highlighted by their ability to act as multipotent mesenchymal stem cells. *In vivo,* pericytes have been shown to differentiate into smooth muscle cells and myofibroblasts, and *in vitro* to osteoblasts, adipocytes and chondroblast [4], [5].

Based on studies on human pathological conditions and animal models, we and others have proposed that pericytes in inflammatory conditions including wound healing in adult tissues become activated and expand into pro-fibrotic connective tissue cells, indicating that these microvascular cells play a central role in tissue fibrosis [6]–[13]. We have developed methods to isolate and propagate pericytes from placenta and neonatal skin which has enabled the study of pericyte biology and in particular their differentiation into collagen type I producing fibroblasts, a process that spontaneously takes place when pericytes are cultured in the presence of 10% fetal calf serum (FCS) [6]–[9].

Epigenetic inheritance represents heritable patterns in gene expression caused by mechanisms other than changes in underlying DNA sequences [14]. Thus, non-genetic factors can cause genes to behave differently opening up possibilities such as modifying the phenotype of congenital and acquired diseases [14]. Furthermore, epigenetic mechanisms are believed to modify the progeny of stem/progenitor cells as well as affect the phenotypes of individual specialized cells and maintain these phenotypes over several rounds of cell division thus constituting a mechanism by which cells have “memory” [15], [16]. The molecular basis of epigenetics is complex but includes DNA methylation and post-translational modifications of histones such as acetylation and methylation. These DNA and histone modifications affect chromatin structure, thereby regulating gene expression [17]. The state of histone acetylation is a balance between reciprocal enzymes, histone acetyltransferases (HATs) and histone deactetylases (HDACs), acetylating and deacetylating histones, respectively [17]. Acetylation generally leads to transcriptionally active chromatin while deacetylation results in transcriptional silencing [18], [19]. Valproic acid (VPA) is an aliphatic acid compound that has been used for decades as a medication to treat epilepsy and as a mood stabilizer in bipolar disorder [18], [20]. VPA is one among several HDAC inhibitors [21], [22]. VPA is also recognized for its teratogenic effects in humans [22], [23].

HDAC inhibition results in hyperacetylation of histones [20] and affects gene expression, which in turn influences cellular proliferation, differentiation, apoptosis and migration [18], [20] as well as more complex multi-cellular processes such as angiogenesis [24]–[29] and fibrosis [25], [30]–[32]. Thus, HDAC inhibition has therefore been postulated to affect genetic perturbations as occurs in for instance congenital and neoplastic disease [17], [20]. Studies have shown potential beneficial effects of HDAC inhibition in a wide array of diseases including cystic fibrosis, malignancies, myelofibrosis and neurodegenerative diseases such as Alzheimers and Huntingtons disease [17], [20].

Several *in vitro* and *in vivo* studies have shown that HDAC inhibition is anti-angiogenic [24]–[29]. The present *in vitro* study is the first investigation into the biological consequences of HDAC inhibition in well-defined primary human microvascular pericytes. HDAC inhibition reduced pericyte proliferation and inhibition of migration without significantly affecting cell viability. The differentiation of pericytes into pro-fibrotic connective tissue cells was reduced by VPA suggesting a novel pericyte based process of how HDAC inhibition may perturb fibrosis. Furthermore, a limited qPCR microarray approach focusing on mRNAs coding for proteins involved in angiogenesis was performed in freshly isolated human pericytes revealing expression patterns of angiogenic mRNAs and the potential effect that HDAC inhibition may have on these expression patterns. The overall results in the present study suggest that HDAC inhibition of pericytes promotes a phenotype involved in vessel stabilization/maturation [24]–[29].

## Results

### VPAs effect on cytotoxicity, cell viability and histone 4 acetylation in human pericytes

**Figure 1:**
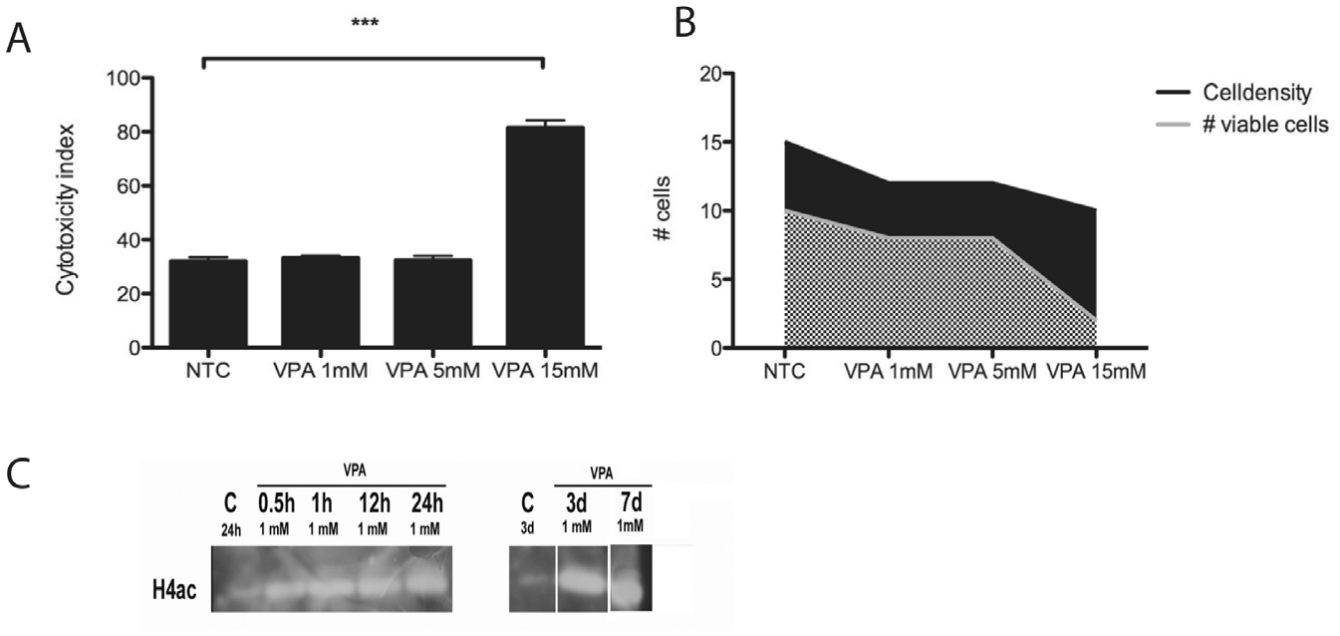
Cytotoxic effect of VPA on human pericytes. (**A**) Cytotoxicity index based on nuclear fragmentation and lysosomal pH in human pericytes exposed to 1–15 mM VPA for 3 days. (**B**) Cell density and viable cell measurement in human pericytes exposed to 1–15 mM VPA for 3 days. (**C**) VPA effects on histone 4 acetylation (H4ac) in human pericytes exposed for different time periods to 2 mM VPA. Mean ± standard deviation (SD), n = 4. T-test * *p*<0.05, ** *p*<0.01, *** *p*<0.001.

Microvascular pericytes with a phenotype consistent with pericytes *in vivo* were isolated from full-term human placentas as previously described [6]–[8]. To investigate pericyte sensitivity to VPA, a characterization of cell cytotoxicity and viability/density was performed. Cytotoxic effect was observed when pericytes were exposed to concentrations of VPA exceeding 5 mM (Figure 1A; t-test p<0.001). A reduction in the proportion of viable cells compared to cell density was also first observed at these concentrations (Figure 1B). Subsequent exposures were therefore conducted at lower concentrations.

VPA has been reported to inhibit HDAC and thereby increase histone acetylation in several cell types [21], [22]. To determine if VPA had HDAC inhibitory effects in pericytes, we assessed the levels of acetylated histone H4 in pericytes exposed to 1 mM VPA and non-treated control (NTC) pericytes. NTC pericytes displayed weak histone H4 acetylation, whereas levels of acetylation were increased already at 0.5 hours and were maintained for up to 7 days in pericytes continuously exposed to 1 mM VPA (Figure 1C).

### Effect of HDAC inhibition on proliferation and cell viability in human pericytes

**Figure 2:**
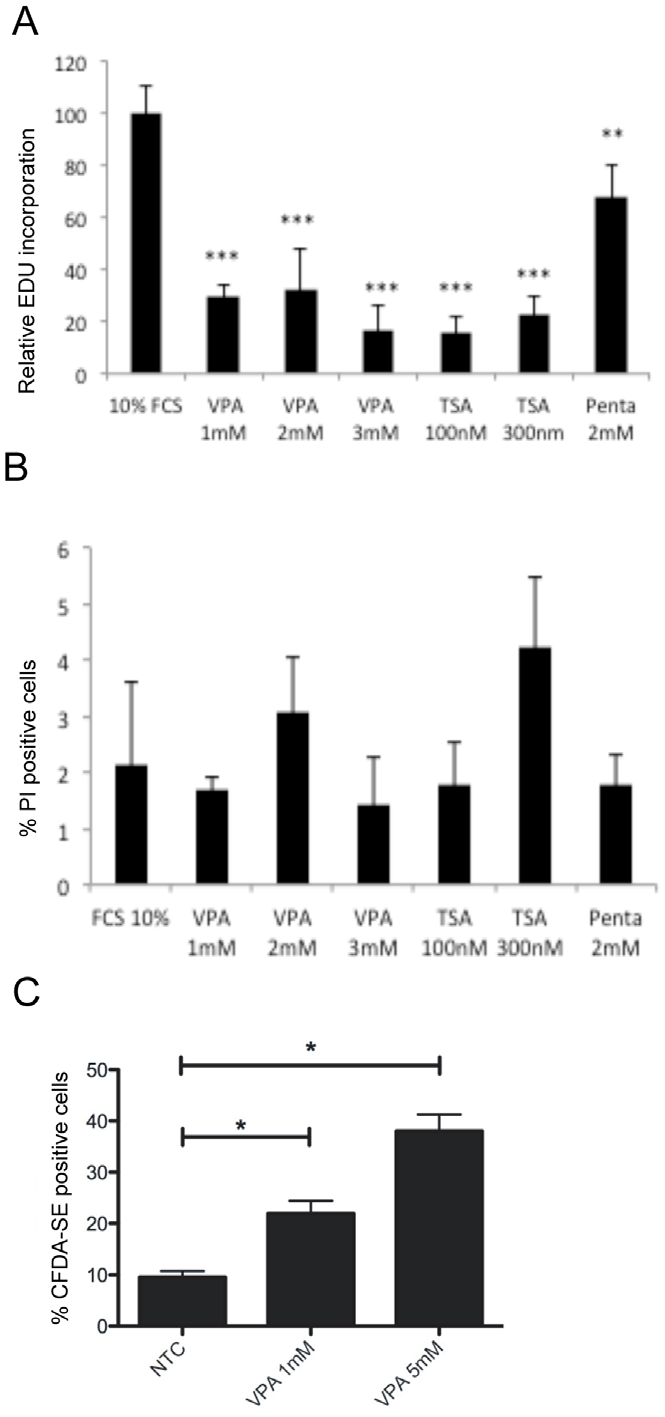
Effect of VPA on human pericyte proliferation and cell viability. (**A**) Proliferation rate *i.e.* % of cells entering S-phase depicted by EDU incorporation in pericytes exposed to VPA, TSA or Penta for 24 hours compared to NTC pericytes (100%). (**B**) Cell viability *i.e.* % of cells positive for PI in pericytes exposed to VPA, TSA or Penta for 24 hours compared to NTC pericytes. (**C**) Flow cytometric analysis of CFDA-SE labeled pericytes exposed to 1 and 5 mM of VPA for 7 days compared to NTC. CFDA-SE retention reflects a lower rate of proliferation in pericytes. Mean ± SD, in A and C n = 6, in B n = 4. T-test * *p*<0.05, ** *p*<0.01, *** *p*<0.001.

We investigated if VPA is able to influence pericyte proliferation. EdU incorporation as a measure of cells entering the S-phase of proliferation was significantly reduced by approximately 70% in pericytes after a 24 h exposure to 1 mM VPA compared to NTC pericytes (Figure 2A; t-test p<0.001). Similar results were achieved when using ^3^H-tymidine incorporation, also an indicator of cells entering the S-phase of proliferation (data not shown). No significant further reduction in pericyte proliferation was observed using higher concentrations of VPA. The reduction in pericyte proliferation and viability when exposed to Trichostatin A (TSA), another compound that inhibits HDAC, was of a similar magnitude when compared to VPA. 2-Et-4-Me-Penta (Penta) is a VPA analogue, which has no inhibitory effect on HDACs [33]–[35]. When pericytes were exposed to Penta there was a statistically significant reduction of pericyte proliferation by approximately 30% compared to NTC pericytes (t-test p<0.01). The reduction of proliferation in pericytes exposed to VPA or TSA was more pronounced than that seen in cells exposed to Penta (Figure 2A).

Previous studies have shown that HDAC inhibition leads to an increase in cell death [18], [20]. Propidium iodide (PI) was used as an indicator of cell viability to investigate if HDAC inhibition of pericytes affected the number of viable cells. No significant differences in the percentage of dead/dying cells were observed when pericytes were exposed for 24 h (Figure 2B) and 72 h (data not shown) to VPA, TSA or the VPA analogue Penta when compared to NTC pericytes.

To investigate the effects of VPA on pericyte proliferation over several cell cycles, cells were labeled with CFDA-SE and further cultured in the presence or absence of VPA for one week. At the 7-day time point CFDA-SE retention was measured by flow cytometry. The principle of the CFDA-SE assay is that the amount of CFDA-SE is distributed evenly between daughter cells following each generation/cell-division [36]. This analysis demonstrated that the percentage of cells retaining CFDA-SE was significantly increased in pericytes exposed to 1 mM and 5 mM VPA at the 7-day time point compared to NTC (Figure 2C; t-test p<0.05).

### VPAs effect on migration of human pericytes

**Figure 3:**
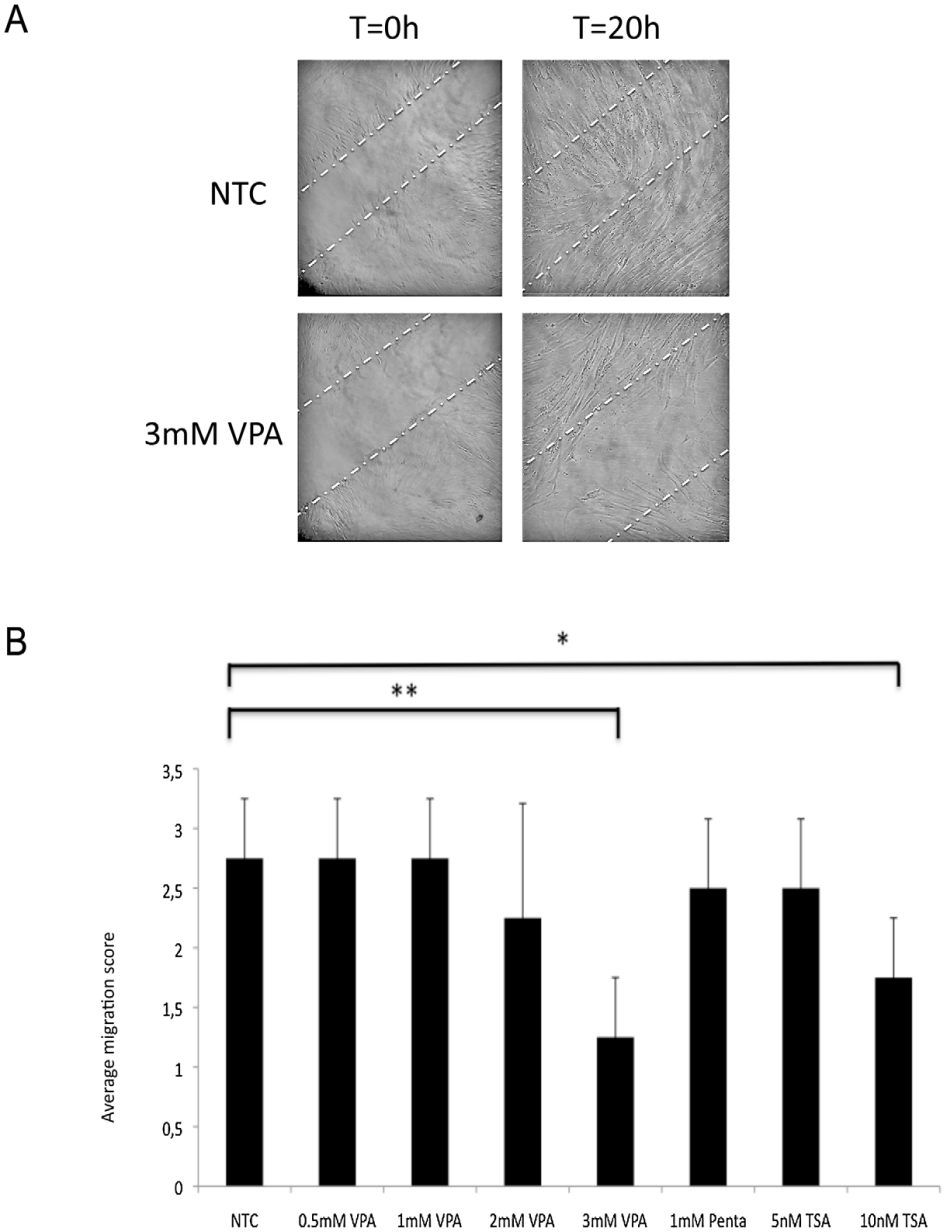
Effect of VPA on human pericyte migration. Effect of exposure of different concentrations of VPA on human pericyte migration in a wound scratch assay was studied. (**A**) Representative images of pericyte cultures in the wound scratch assay at t = 0 and at t = 20 h (white dotted lines designate the borders of the wound scratch), exposed to 2 mM VPA and NTC pericytes. (**B**) Migration scores calculated as outlined in materials and methods were determined in pericyte cultures exposed to different concentrations of VPA, TSA or Penta. Mean ± SD, n = 4. T-test * *p*<0.05, ** *p*<0.01, *** *p*<0.001.

The effect of VPA on migration of pericytes was investigated in a 20 h wound scratch cell migration assay [37] (Figure 3A). The migration score was quantified as outlined in Materials and Methods. Migration was significantly inhibited when pericytes were exposed to 3 mM VPA (t-test p<0.01). No significant effect on pericyte migration was observed at lower VPA concentrations (Figure 3B). The VPA analogue Penta lacking HDAC inhibitory activity had no significant effect on pericyte migration (Figure 3B). Pericytes exposed to 10 nM TSA, a more potent HDAC inhibitor, was significantly reduced compared to NTC pericytes (Figure 3B; t-test p<0.05). This effect was not seen at 5 nM TSA.

### VPAs effect on differentiation of human pericytes to collagen type I producing fibroblasts

**Figure 4:**
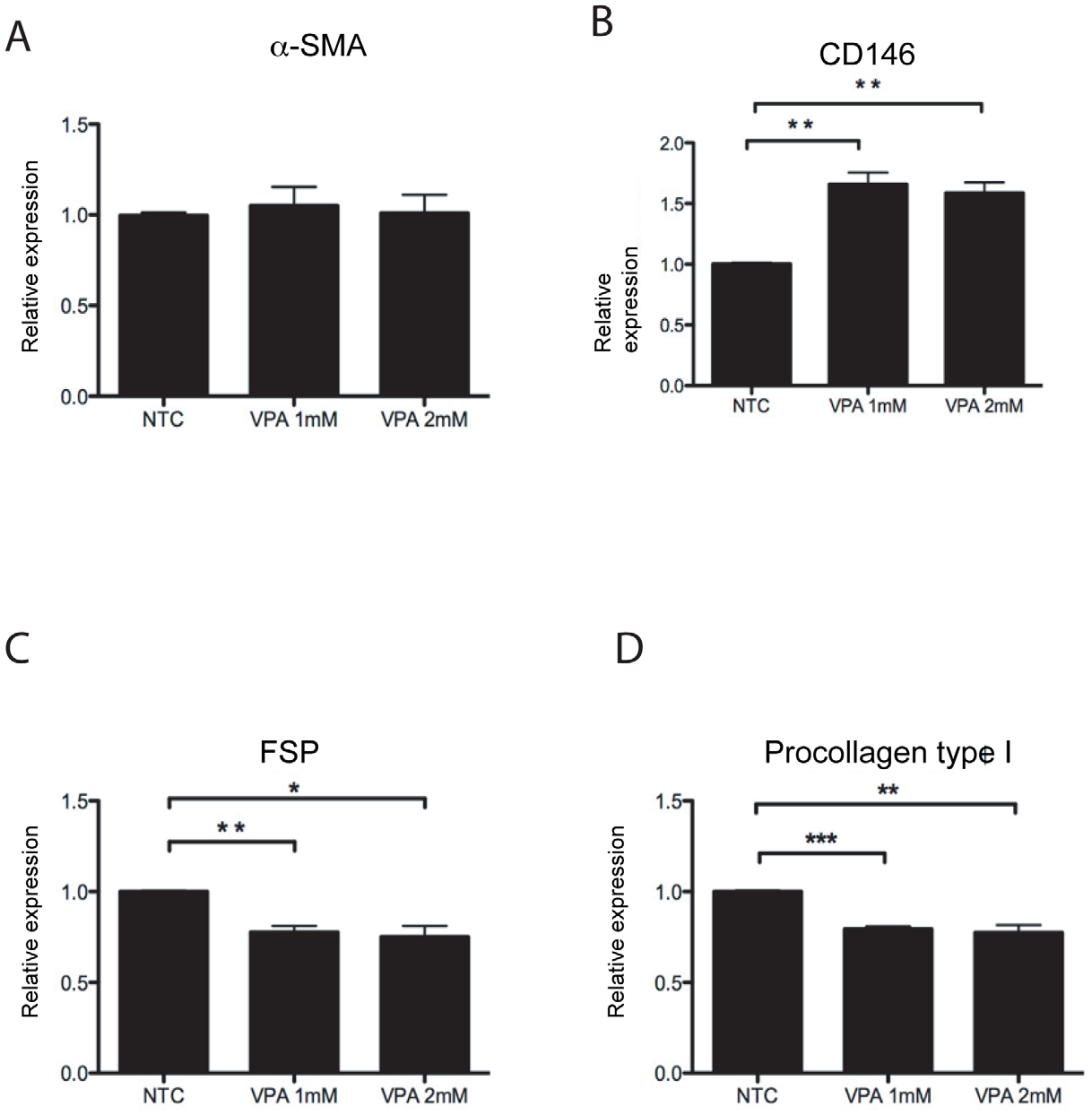
Effect of VPA on human pericyte differentiation. Quantification by flow cytometry, displayed as “relative expression” compared to NTC pericytes (1.0) within “sets” of 10.000 analyzed cells, of protein markers that define different stages of the differentiation process from microvascular pericyte to collagen type I producing fibroblast in pericyte cultures exposed to 1 mM and 2 mM VPA compared to NTC. (**A**) α-SMA, (**B**) CD146, (**C**) FSP, (**D**) procollagen type I. Mean ± SD, n = 3. T-test * *p*<0.05, ** *p*<0.01, *** *p*<0.001.

We have previously reported that pericytes differentiate into procollagen type I producing fibroblasts in 10% FCS culture conditions and that this differentiation process can be monitored by the expression patterns of several protein markers [6]–[8]. The relative expression of the number of cells expressing each marker was quantified by flow cytometry and compared to NTC pericytes (Figure 4A–D). VPA exposure at a concentration of 1 or 2 mM, during a 72-hour incubation period inhibited this differentiation process based on the observed shift in marker expression profiles compared to NTC pericytes (Figure 4A–D). The relative number of cells expressing α-SMA, a marker expressed in both pericytes and fibroblasts [9], [38]–[40], were not affected by VPA exposure (Figure 4A). VPA exposure significantly increased (t-test p<0.01) the relative number of cells expressing the mesenchymal stem cell marker CD146 (M-CAM) [41], [42] (Figure 4B). A significantly reduced number of cells expressed the fibroblast surface protein” (FSP) [43] (Figure 4C; t-test p<0.01) and procollagen type I [41]–[43] (Figure 4D; t-test p<0.001) after VPA exposure.

### VPAs effect on mRNA expression of genes involved in angiogenesis in human pericytes

**Figure 5:**
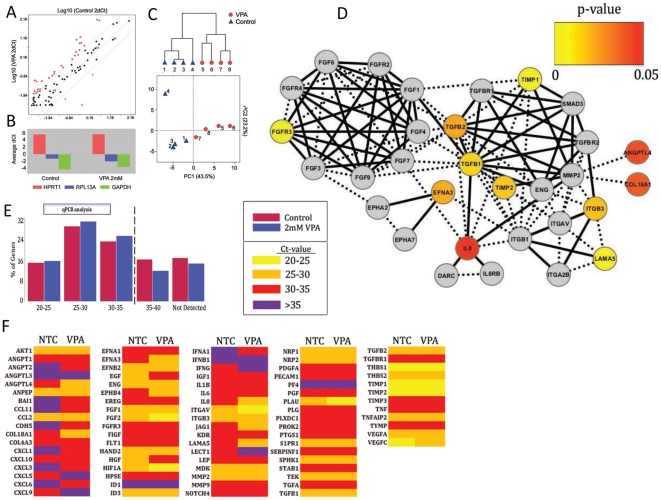
Effect of VPA on expression of angiogenesis related genes in human pericytes. Characterization of changes in angiogenesis gene expression in pericytes exposed to 2 mM VPA over three days compared to NTC using a qPCR angiogenesis microarray. (**A**) Scatter plot based on transformed ΔCt values for NTC and VPA for each gene. Each axis is in the log^10^ scale. The letter H represents the expression of housekeeping genes. (**B**) The average ΔCt value for the three housekeeping genes (HPRT1, RPL13A and GAPDH) used in the qPCR analysis. (**C**) Combination of unsupervised hierarchical clustering and principal component analysis. The dendrogram shows that NTC and VPA samples separate from each other. The first component (PC1) explains 43.5% of the variation in the data and separates NTC from VPA samples. NTC sample 4 differs somewhat from the other NTC (see PC2). (**D**) Network analysis of the most significant angiogenesis genes using data exported from the STRING database and visualized in Cytoscape. Edges between nodes are based on different levels of evidence. Solid lines are based on a high combined STRING confidence level (>0.9) whereas dotted lines are <0.9. The color of the nodes/genes is based on their p-value from the pair-wise comparison between NTC and VPA samples. Gray colored nodes represent genes not analyzed in this experiment and do therefore not possess any p-values. (**E**) Percentage of the total included 84 genes that were expressed in each Ct value range in VPA and NTC pericytes. (**F**) Shifts in Ct value ranges in each individual gene in VPA compared to NTC pericytes.

Considering the role of pericytes in angiogenesis, we next analyzed the effect of VPA on a group of angiogenesis related genes after three days of exposure to 2 mM VPA. Pericytes derived from four separate placentas were analyzed with qPCR angiogenesis arrays (Figure 5, for a list of abbreviations see Table S1). A total of 14 genes (∼18%) were significantly affected in pericytes exposed to VPA compared to NTC pericytes (Table 1; t-test p<0.05). An analysis of the data shows that VPA exposure in general increases gene expression of angiogenesis associated genes in pericytes (Figure 5A). Only 4 genes showed a tendency towards down regulation but did not reach statistical significance (data not shown). The data was normalized against three housekeeping genes HPRT1, RPL13A and GAPDH (Figure 5B). A clear separation between the VPA exposed pericytes and NTC pericytes can be seen in the principal component analysis (PCA) (Figure 5C). The top significant genes were searched against the protein interaction database STRING version 8.3 (http://string.embl.de/) for protein-protein interaction networks and the results visualized using the Cytoscape software (Figure 5D). STRING interactions are based on different sources such as annotated experimental data, co-expression, and text mining. A high STRING interaction score (1.0 being the maximum score) between genes/proteins correlates with multiple sources of evidence. This analysis identified additional genes/proteins, which were not included in the qPCR angiogenesis array but are known to interact with the proteins, which mRNAs were significantly altered. This analysis identified two potential separate protein interaction clusters. The interaction analysis indicates that a number of the significant VPA induced genes are directly or indirectly connected to a TGF-β and TIMP associated network, which themselves were significantly upregulated (Figure 5D and Table 1). In NTC pericytes, of the 84 genes studied, a relatively large proportion (36 genes ∼43%), included in the qPCR angiogenesis array had Ct values >35 (14 genes ∼17%; Figure 5E and F) which is the cut-off limit recommended by the manufacturers, or were not detected at all (22 genes ∼26%; Figure 5E and F). In pericytes exposed to VPA compared to NTC pericytes 13 genes (∼15%) were induced *i.e.* had a CT value of less than 35 when exposed to VPA (Figure 5F). Only one genes expression (PDGF-A) was extinguished by VPA exposure.

**Table 1:** Upregulation of angiogenesis related genes in human pericytes exposed to 2 mM VPA.

Gene	Protein	p-value	VPA v. NTC
ANGPTL4	Angiopoietin-like 4	0.042	46.76
EFNA3	Ephrin-A3	0.022	25.35
FGFR3	Fibroblast growth factor receptor 3	0.003	21.56
PTGS1	Prostaglandin-endoperoxide synthase 1	0.012	17.52
IL8	Interleukin 8	0.049	10.3
TYMP	Thymidine phosphorylase	0.0005	10.06
ITGB3	Integrin beta3 (CD61)	0.018	9,55
TGFB2	Transforming growth factor beta2	0.023	9.07
TIMP1	TIMP metallopeptidase inhibitor 1	0.00006	8.41
LAMA5	Laminin alpha5	0.008	4.56
PLXDC1	Plexin domain containing 1	0.031	4.33
COL18A1	Collagen type XVIII alpha1	0.039	3.34
TGFB1	Transforming growth factor beta1	0.014	3.34
TIMP2	TIMP metallopeptidase inhibitor 2	0.016	2.57

Fold regulation of the expression of genes significantly altered in pericytes exposed to 2 mM VPA for 3 days compared to NTC. *P-value* <0.05 was considered significant.

## Discussion

The aim of this study was to probe if HDAC inhibition has any effect on human microvascular pericytes with regards to proliferation, cell viability, migration and differentiation into pro-fibrotic connective tissue cells *in vitro*. Furthermore, given the growing importance of the role of the pericyte in the regulation of the vasculature and the process of angiogenesis focus was directed towards potential modifications of expression patterns of mRNAs coding for proteins known to be involved in the angiogenic process in pericytes in response to HDAC inhibition.

The effect of HDAC inhibition on pericyte proliferation was investigated. Inhibition of pericyte proliferation was seen already at a concentration of 1 mM VPA. Although the VPA analogue Penta which lacks HDAC inhibitory effects had a statistically significant effect on pericyte proliferation the effect did not approximate that observed in pericytes exposed to VPA suggesting that the anti-proliferative effect was to a large extent due to VPAs ability to inhibit HDACs. However, a certain effect on pericyte proliferation due to the “off target effects” of VPA cannot be excluded. The notion that it is HDAC inhibition that is responsible for the effects seen on pericyte proliferation were further supported by experiments using TSA, another more potent HDAC inhibitor, which had similar anti-proliferative effects on pericytes when compared to VPA. The CFDA-SE incorporation experiments show that VPA, in a concentration dependent manner, limits the expansion of the pericyte population over the course of several cell divisions [36], [44], and resulted in a higher percentage of pericytes retaining the dye. Previous studies addressing the potential role of HDAC inhibitors in blood vessel biology have focused on the endothelium [24]–[29]. These *in vitro* results show that HDAC inhibition has an anti-proliferative effect also on pericytes and limits the expansion of the pericyte population over time.

The present investigation shows that HDAC inhibition perturbs pericyte migration. When analyzing results from migration assays cell proliferation and viability must be taken into account in that a decrease in proliferation/viability may be misinterpreted as an inhibition of migration. In the present investigation no significant changes in the number of viable cells was seen after exposing pericytes to the concentrations of VPA used in the migration assay. Thus, cell death should not influence the observed results. At the 24-hour time point inhibitory effects on proliferation, but not migration, were seen in pericytes exposed to 1 mM VPA. VPAs inhibitory effect on migration was first significant in pericytes exposed to 3 mM VPA. Thus, a decrease in pericyte proliferation should not influence the observed results. The HDAC inhibitor TSA had a similar effect on pericyte migration. No effect on migration was observed when cells were exposed to the VPA analogue Penta suggesting that HDAC inhibition and not “off target effects” of VPA lead to the observed inhibition of pericyte migration. The results show that HDAC inhibition inhibits pericyte migration.

The present investigation shows that VPA induces and upregulates expression of mRNAs coding for proteins involved in angiogenesis in pericytes *in vitro*. HDAC inhibition has been shown to inhibit angiogenesis *in vivo* and endothelial cell proliferation *in vitro* [24]–[29]. No studies to our knowledge have addressed the role of pericytes in HDAC mediated effects on angiogenesis. Several pathological states in adults are initially characterized by an excessive and aberrant angiogenesis. Vascular regression and pruning followed by vascular maturation is required for the finalization of tissue reparative processes [45]–[48]. The qPCR data support that VPA induces a phenotype in pericytes consistent with events that occur during the later stages of angiogenesis *i.e.* vessel stabilization and maturation. Several of the genes that were upregulated in pericytes exposed to VPA are involved in endothelial cell survival (angiopoietin like 4 [49], [50] and IL-8 [51], [52]), endothelial tube formation/stabilization/branching (FGFR-3 [53]–[55] and integrin β3-subunit [56]), formation and maintenance of direct cell-cell contacts between endothelial cells and pericytes (TGF- β1 and 2 [57]–[59]), basement membrane formation (laminin α5 [60] and collagen type 18 [45], [57]) and inhibition of metalloprotease activity (TIMP 1 and 2 [61]). All of these events are consistent with vessel stabilization/maturation [45]. A further support for this interpretation is that genes only detected in pericytes exposed to VPA *i.e.* expression was induced by VPA, included gene products involved in endothelial survival, differentiation and fenestration (prokineticin 2 [62], [63] and stabilin-1 [64]) as well as angiostasis (Cxcl3 and 6 [65], [66] and epiregulin). Thus, we hypothesize that the observed inhibition of proliferation and migration together with the mRNA expression profile in response to VPA results in a pericyte phenotype consistent with vessel stabilization/maturation.

The present investigation shows that exposure to VPA inhibits differentiation of pericytes into pro-fibrotic connective tissue cells. We have in previous studies demonstrated that pericytes with a phenotype similar to that seen *in vivo* can be maintained *in vitro* [6]–[8]. Increasing evidence suggest that pericytes are capable of differentiating into collagen type I producing fibroblasts both *in vitro* and *in vivo*, thereby implicating the microvascular pericyte in the process of fibrosis in a previously unrecognized way [6]–[13], [67]–[70]. This differentiation process was studied *in vitro* by examining shifts in the expression of markers over time by flow cytometry [6]–[8]. VPA increased the proportion of cells with a marker expression profile consistent with the pericyte phenotype while decreasing the proportion of cells with a marker profile expression characteristic of fibroblasts. The results show that VPA inhibits the differentiation of pericytes into pro-fibrotic collagen type I producing fibroblasts. In support of this notion the proportion of cells expressing procollagen type I a marker for collagen type I synthesis was decreased in pericytes exposed to VPA. Previous *in vitro* and *in vivo* studies in for instance liver, pancreas and kidney have shown that HDAC inhibition is anti-fibrotic by reducing the proliferation and activation of pro-fibrotic connective tissue cells and the production of collagen type I [25], [30]–[32], [71]–[75]. However, the coupling of the anti-fibrotic effects of HDAC inhibition to microvascular pericytes has not previously been recognized. VPA is the first compound that has been identified as being able to inhibit this differentiation process. We speculate that the current results may in part explain the anti-fibrotic effects of HDAC inhibition observed *in vivo*. However, future studies will be required to address this issue.

The results suggest that HDACs may have a dual role in pericyte behaviour. HDAC inhibition leads to appropriate maturation/stabilization of vessels on the one hand and inhibition of the development of pro-fibrotic connective tissue cells on the other. Interestingly, gene deletion studies in mice show that PDGF and their receptors while important for the expansion of the pericyte population during embryogenesis is crucial for expansion of the fibroblast population in reactive connective tissue formation in adults suggesting a common origin of these two cell-types [68], [76]. Thus, the role of pericytes during embryogenesis compared to wound healing processes in adults may be quite different. We speculate that it would be advantageous for pericytes to behave more “embryonic” in order to optimally generate and regenerate tissues in the adult animal. Furthermore, that inhibiting the driving forces that regulate pericyte differentiation to collagen type I producing fibroblast will have a positive influence on limiting fibrosis. Thus, modulating HDAC function may have a role in both of these processes by affecting pericytes. In conclusion HDAC inhibition through VPA affects microvascular pericyte function and phenotype and may have important ramifications in limiting fibrosis while promoting tissue repair/regeneration following tissue injury secondary to trauma and/or pathology. Future *in vivo* studies using different types of HDAC inhibitors in pathologies as well as models of tissue regeneration/repair will be required to further elucidate the dual effect of HDAC inhibition on pericytes and the potential for optimal tissue repair/regeneration with minimal fibrosis.

## Materials and Methods

### Cell isolation and cell culture

Primary pericyte cultures from human placenta were isolated as described previously [6] and maintained in growth media: RPMI 1640 (Gibco, CA, USA) containing 10% FCS (Saveen-Werner, Sweden) and Penicillin/Streptomycin (SVA, Uppsala, Sweden). All cell culture incubations were performed at 37°C in humidified incubators with 5% CO_2_.

### Ethics Statement

The acquisition of human placentas was approved by the human ethics committee of the University of Uppsala, Sweden. The placentas were anonymous and there was no personal data collected on the donors.

### Cytotoxicity

For cytotoxicity studies 5 000 pericytes per well were seeded onto collagen type I (Vitrogen, CA, USA) coated (50 µg/ml for >1 h) 96-well plates (Perkin-Elmer Inc. Wellesley, MA, USA) and grown for 18 h before the addition of 1, 5 or 15 mM of VPA (P4543, Sigma Aldrich, Sweden). RPMI 1640 containing 10% FCS was used as a control (NTC). Plates were incubated for 72 h before the cells were stained with the Multiparameter Cytotoxicity 1 Kit (Cellomics Inc., PA, USA). Plates were read with the ArrayScan high content screen system (Cellomics Inc.) and a cytotoxicity index based on nuclear morphology/size, cell membrane permeability, lysosomal mass-pH and cell density, was obtained according to the manufacturers instructions.

### Proliferation

Pericytes were seeded in 60 mm plates (Sarstedt, Germany) and incubated for 24–48 hours in RPMI growth media. One day prior to harvesting pericytes were exposed to different concentrations of trichostatin A (TSA) purchased from Sigma Aldrich, VPA, and the VPA analogue Penta (a kind gift from Heinz Nau at the University of Veterinary Medicine, Hannover, Germany) or growth medium with vehicle (DMSO). Four hours prior to harvesting 10 µM 5-ethynyl-2′-deoxyuridine (EdU), used as a proliferation marker, detecting cells entering the S-phase of the cell cycle was added to the cells. 10 minutes prior to harvesting 2.5 µg/ml propidium iodide (Invitrogen, Eugene, Oregon, USA), used as a marker for cell viability, was added. Cells were then washed, fixed in 4% paraformaldehyd and processed according to the manufacturers protocol for the “Click-iT EdU Pacific Blue Flow Cytometry Assay Kit” (Invitrogen). Cells were then analyzed by flow cytometry using a FACSAria (BD Biosciences, Sweden).

### CFDA-SE assay

In the Carboxyfluorescein diacetate succinimidyl ester (CFDA-SE) proliferation assay, cells are loaded with CFDA-SE that spontaneously and irreversibly bind to cellular proteins by reacting with lysine side chains and other available amines [36], [44]. Cells are then seeded into culture plates and for each cell division that takes place CFDA-SE is distributed equally between the daughter cells, which therefore have half the fluorescence intensity compared to the parent cell. Thus, for each successive generation in a population of proliferating cells a two-fold decrement in cellular fluorescence intensity occurs that can be determined by flow cytometry. The proliferating cell population can then be studied over time and the number of cell divisions that have taken place can be calculated allowing for cells to be followed over several generations. Passage two pericytes were trypsinized and collected in a pellet. The pellet was resuspended in RPMI 1640 containing 0.5 µM CFDA-SE and left at 37°C for 10 minutes. RPMI 1640 containing 10% FCS was added and cells were centrifuged at 200 rpm for 5 minutes. Cells were seeded in 6 well plates in RPMI 1640 containing 10% FCS and 1 or 5 mM VPA. RPMI 1640 containing 10% FCS with and without VPA was used as control. After seven days of VPA exposure cells were trypsinized and collected and analyzed by flow cytometry for the expression of CFDA-SE.

### Immunoblotting

Pericytes were cultured in 8-well plates with or without 1 mM VPA for 30 min, 1 h, 12 h, 24 h, 3 d and 7 d. The cells were solubilized in RIPA buffer (150 mM NaCl, 1% NP-40, 0,5% deoxycholic acid, 0,1% SDS, 50 mM Tris pH 8.0) and protein concentrations were quantified using the 2D-Quant kit (GE Healthcare, Uppsala, Sweden). Western blot using an H4K5/8/12/16ac antibody (dilution 1∶3000) (06-866, Upstate Biotechnology) was performed using the ECL Advance western blotting detection system (GE Healthcare) according to the manufacturers guidelines. Briefly, 10 µg of the protein samples were separated by the BioRad mini-Protean system on a 12.5% SDS-PAGE gel using Laemmeli buffer (25 mM Tris, 192 mM Glycine and 0,1% SDS) as a running buffer. The proteins were then transferred to 0.45 µm nitrocellulose membrane (Hybond ECL, GE Healthcare) in a blotting cassette (BioRad mini-Trans-Blot cell) followed by incubation with the primary antibody and then the secondary antibody (Santa Cruz, CA, USA) conjugated to horseradish peroxidase (HRP). The immunoblots were exposed to Cronex 5 light sensitive film.

### Migration assay

Cells were seeded into 24-well plates at 20 000 cells/cm^2^ and were allowed to attach for 12 h in RPMI 1640 containing 10% FCS. A uniform scratch, using a 200 µl pipette, was afflicted in the middle of each well creating an area devoid of cells (Figure 3A). These pericyte cultures were then exposed to RPMI 1640 medium containing 10% FCS and VPA, TSA or Penta or vehicle for 24 hours. RPMI 1640 medium containing 10% FCS without VPA was used as control. Semi-quantifications of the extent of pericyte migration into the cellular gap/void over time were performed. Each treatment group was observed in four replicates. The individual migration score can either be 0 (no migration at all), 1 (migration initiated along the border but pericytes do not spanning the gap/void), 2 (migrating pericytes are spanning the gap/void but not completely populating the gap/void), and 3 (migrating pericytes have completely repopulated the gap/void) [37]. The average score of replicates/groups is calculated and compared.

### Differentiation

Passage two pericytes were seeded in 6-well plates and exposed to 1 or 2 mM VPA in RPMI 1640 containing 10% FCS for 72 hours. RPMI 1640 containing 10% FCS was used as controls. After 72 hours cells were trypsinized and labeled with antibodies against the pericyte marker CD146 (mAb24577, Abcam, UK) [41], [42]; the fibroblast markers; Fibroblast surface protein (mAb FSP) (F771, SigmaAldrich) [43] and procollagen type I (pAb64409, Abcam, United Kingdom); and α-smooth muscle actin (mAb α-SMA, F3777, Sigma Aldrich) a marker expressed by both pericytes and fibroblasts [9], [38]–[40]. Cells were labeled in PBS containing 0.2% BSA for 20 minutes on ice, washed twice with PBS containing 0.2% BSA before a 20 minute incubation on ice with the secondary antibody diluted in PBS containing 0.2% BSA (Allophycocyanin-goat anti mouse IgG (115-136-072, Jackson Immuno Research, UK), FITC-goat anti mouse IgM (115-096-075, Jackson Immuno Research) or FITC-rat anti mouse IgG (FI-4000, Vector Laboratories Inc, CA, USA). Cells were then washed two times before being analyzed by flow cytometry on a FACSAria flow cytometer (BD Biosciences). Isotype Mouse IgG (I-8765, Sigma Aldrich, Sweden), IgM (ab 18401, Abcam, UK), and rat IgG were used as controls and to determined background levels of staining intensity. Based on controls cells were either positive or negative for the expression of each marker. Several sets of 10,000 cells were analyzed. The relative expression *i.e.* the number of cells positive for each marker was determined and compared between the different conditions. Experiments were performed on cells isolated from three different placentas.

### Angiogenesis quantitative PCR array

RNA was extracted from cultured pericytes after three days of culture in 24-well plates (starting with ∼3×10^5^ cells per well) in RPMI 1640 medium containing 10% FCS with or without 2 mM VPA. The cells were washed twice with cold PBS before being lysed directly in the well with β-mercaptoethanol containing lysis buffer provided in the RNeasy Mini kit (QIAGEN, Denmark), followed by the use of QIA shredder spin columns (QIAGEN). Subsequent RNA preparation was done according to the protocol for the RNeasy Mini kit. RNA concentration was determined by the use of a NanoDrop ND-1000 Spectrophotometer (Nanodrop Technologies, DE, USA), and quality control was done with the Experion system and RNA StdSens chips (Bio-Rad, CA, USA). cDNA synthesis based on 0.33 µg total RNA per sample was performed using the RT^2^ First Strand Kit (SA Biosciences, MD, USA). Real-time PCR was performed on an iCyclerIQ (Bio-Rad) using the RT^2^ qPCR SYBR green mastermix and Human Angiogenesis RT^2^ Profiler PCR 96-well Arrays (Catalogue number PAHS-024A, SA Biosciences) [77] and the recommended PCR cycling program (10 min 95°C (1 cycle), 15 s 95°C plus 60 s 60°C (40 cycles)) [77]. See gene symbols and their corresponding descriptions in Table S1. The total volume of the PCR was 25 µl. Samples were run in a randomized order to prevent any technical and design bias. The PCR array data passed the SA Biosciences online quality control. Data analysis of relative gene expression was performed using the ΔΔC_t_ method with HPRT1, RPL13A and GAPDH as housekeeping genes. Fold change values and Students t-test p-values were exported from SA Biosciences PCR array analysis web service (www.sabiosciences.com/pcrarraydataanalysis.php). ΔC_t_ values were exported from the SA biosciences PCR array data analysis web portal for further analysis. General properties in the data set were visualized using hierarchical clustering (complete linkage and Euclidian distance) in the R software (version 2.11). Top candidate genes were searched against the STRING protein-protein interaction database (version 8.3, http://string.embl.de/). The resulting network data was expanded with the addition of 10 more gene/protein partners known to interact with the top candidates but not part of the qPCR genes to connect separate clusters. Network data was exported and visualized in the Cytoscape software. Interaction score strength based on STRING criteria was defined as either >0.9 or <0.9 (1.0 being the top score). The analysis is based on the isolation of pericytes from 4 different placentas

### Data analysis

All data are shown as mean ± SD. Comparisons between two groups were analyzed using unpaired Students *t*-test. A *P* value <0,05 was considered to be statistically significant. All experiments were performed at least in triplicates.

## Supporting Information

Table S1 **Gene symbols and gene description included in the qPCR array.** Information derived from the manufacturer of the array (Catalogue number PAHS-024A, SA Biosciences).
(DOCX)
